# The association between sleep consolidation and growth and development in early childhood: A systematic review

**DOI:** 10.34172/hpp.43037

**Published:** 2024-12-30

**Authors:** Maryam Bemanalizadeh, Mahan Parsapoor, Leila Emami, Vida Imani, Zahra Parsapour, Roya Kelisahdi

**Affiliations:** ^1^Child Growth and Development Research Center, Research Institute for Primordial Prevention of Non-Communicable Disease, Isfahan University of Medical Sciences, Isfahan, Iran; ^2^Student Research Committee, Shahrekord University of Medical Sciences, Shahrekord, Iran; ^3^Amir Alam Hospital, Tehran University of Medical Sciences, Otorhinolaryngology Research Center, Tehran, Iran; ^4^Pediatric Health Research Center, Tabriz, Iran; ^5^Sleep Disorders Unit, Neurology Department, Acibadem University Medical Faculty, Istanbul, Turkey

**Keywords:** Child, Infant, Growth and development, Sleep, Sleep duration

## Abstract

**Background:**

Achieving sleep consolidation, during the first years of life can be a key factor affecting child growth and development. To our knowledge, for the first time, we aimed to systematically assess the relationships between sleep consolidation and growth and development in early childhood.

**Methods:**

Following the latest version of PRISMA, PubMed, Web of Science, and Scopus were searched up to February 2023. We included observational studies in which 0-3-year-old children were enrolled and the association between sleep consolidation and either children’s growth or development were assessed. The quality assessment was done using the NIH quality assessment tool.

**Results:**

Out of the 342 studies initially screened, 18 studies met the eligibility criteria, encompassing a total of 10068 infants and toddlers under 3 years of age. Overall, not in all but in some studies sleep consolidation showed a significant association with better cognitive, social-emotional and language outcomes. However, the relationship between sleep consolidation and motor development was less clear, with no significant associations observed across the studies. Additionally, no significant connections were found between sleep consolidation and physical growth indices, such as body mass index (BMI) or weight gain.

**Conclusion:**

According to the existing evidence, at least, the potential associations between sleep consolidation and child development particularly cognitive, social-emotional, and language cannot be ruled out. However, due to the heterogenicity of results and inconsistent findings in some studies, we cannot still strongly declare that sleep consolidation is a remarkable predictor for child growth and development.

## Introduction

 Sleep plays an important role in growth, development, and health in the early years of life. Studies have shown that an estimated 20% to 30% of children have some significant night-time waking and bedtime problems.^1,2^ Night-time waking is one of the most common sleep problems in infants. Up to 25% to 50% of children older than 6 months of age also continue to wake up during the night.^3^

 Sleep characteristics change throughout the first year of life. These changes occur due to the maturity of circadian rhythm and the differentiation of the sleep stages through the first 12 months of life.^4,5^ As a result of these changes, night-time sleep duration increases. Infants can sleep for at least 5 hours continuously at night at 12 months of age. It is normal for them to wake up 3-4 times during their night-time sleep but many of them have learned to fall again to sleep on their own.^2,6^ Moreover, in the first 12 months of life, the duration of daytime naps decreases.^7^ The process of the ability of infants to sleep continuously and for longer periods at night while decreasing the time of daytime naps is called sleep consolidation.^8^

 Overall, based on previous studies, sleep is essential for the regulation of various biological processes including the hypothalamic-pituitary-adrenal-axis regulation, which is involved in growth hormone (GH) secretion and release. During slow-wave sleep, there is an increase in GH secretion. This peak of GH during sleep is fundamental for growth and muscle development, as well as tissue regeneration and repair. Therefore, optimal physical growth in early childhood is associated with consistent and consolidated sleep patterns. Furthermore, GH has been identified as one of the potential mechanisms that associate sleep with body composition. Disrupted or fragmented sleep can interfere with these processes, potentially impacting growth and overall health in children.^9^ Sleep is also closely linked to cognitive functions, such as memory consolidation, attention, and problem-solving skills. Thus, those who experience frequent sleep disruptions may struggle with cognitive tasks, which can affect overall brain development. Moreover, sleep plays a key role in emotional regulation and behavior. Adequate sleep can help regulate mood, reduce irritability, and enhance emotional resilience. On the other hand, poor sleep quality has been linked to emotional dysregulation and behavioral problems.^10,11^

 Many studies have investigated the association between sleep consolidation and growth and development in infants and children, but the results have been inconclusive. Some studies, for instance, found that less sleep consolidation and more night awakenings in infants are associated with more behavioral issues, poorer language development, and lower cognitive performance.^12-14^ In contrast, some studies reported no relationship between sleep consolidation and various developmental domains. Pennestri et al conducted a survey on 388 infants between 6 and 12 months and found no association between sleeping 6 or 8 hours uninterrupted at night and their development at 36 months.^15^ Likewise, there are several studies with inconsistent results assessing the relationship between sleep consolidation and growth indices. In a cohort study conducted on 2038 children between 6 and 36 months of age, no association between night awakenings and body mass index (BMI) z-score was detected.^16^ Over the first few months of life, infants develop the ability to retain more calories, so they need fewer calories for growth relative to their size. This allows them to consume adequate calories during the day and progressively need fewer nighttime meals to sustain growth.^17^ Therefore, night feedings are not physiologically necessary for children after 6 months, but sometimes night feedings after 6 months may become a habit that can lead to more night awakenings and reduce the ability of infants to get back to sleep by themselves.^8^

 Since there are conflicting results regarding the effect of sleep consolidation on the growth and development of children, we aimed to conduct a systematic review to investigate the effect of sleep consolidation on developmental domains (i.e. cognitive development, social-emotional development, language, and motor development) and growth indices during the first years of life.

## Methods

 This systematic review was performed following the latest version of the Preferred Reporting Items for Systematic Reviews and Meta-analyses (PRISMA) reporting guidelines.^18,19^

###  Search strategy

 For this systematic review, we found relevant studies by searching and recording through electronic databases including Medline (PubMed), Web of Science, and Scopus from inception until May 2023. A literature search was done using a combination of keywords including “Growth”, “Development”, “Sleep consolidation”, “Night feeding”, and “Night-time waking” and their MeSH equivalents (Supplementary file 1, Table S1). In addition, reference lists of identified studies and review articles as well as Google Scholar were reviewed for potentially missed studies.

###  Eligibility criteria

 Those studies included in the present systematic review met the following criteria: observational studies i.e. cohort, and cross-sectional studies in which children’s development or growth indices associations with sleep consolidation or night awakening were reported. It should be noted that we just included the studies with children equal to or younger than 36 months of age. Based on our preliminary literature review, we included all the studies reporting at least one of the following outcomes: (i) different domains of development including cognitive; social-emotional; language; or motor development (i.e. fine motor and gross motor); as well as (ii) growth indices such as height, weight, or BMI, etc. Sleep consolidation was defined as the process of the ability of infants to sleep continuously and for longer periods at night with decreasing daytime naps (i.e., fewer night wakings and more uninterrupted night sleep).^8^ Studies were screened for duplicate information based on study characteristics. If survey findings were published in multiple articles, only the most recent or comprehensive study was included. Those studies did not report the original results i.e. book chapters, reviews, meta-analyses, commentaries, conference abstracts, letters, and editorials were excluded. We also excluded the studies with a small sample size i.e. case reports and case series.

###  Study selection

 After transferring the search results to a reference manager software (Endnote X9 for Windows, Philadelphia, PA) and removing duplicate records, two researchers (MB and MP) independently screened the titles and abstracts of the retrieved studies. Then, the remaining relevant studies were screened based on their full text using the pre-determined inclusion and exclusion criteria. To reach a consensus, disagreements were discussed with the senior member of the research team (ZP) in each step.

###  Data extraction

 Initially, relevant data were extracted by MB and MP and then checked by ZP. These data were extracted using a prepared checklist consisting of the first author, publication year, study design, sample size, age, sleep consolidation, development or growth outcomes, covariates, and main findings for each included study.

###  Quality assessment 

 Two independent reviewers evaluated the methodological quality of the included studies. We used the 14-item NIH quality assessment tool for observational cohort and cross-sectional studies.^20^ These 14 items were assessed as a judgment with “yes”, “no”, or “other (i.e. cannot determine [CD], not applicable [NA], or not reported^21^)” answers for each question. Finally, the quality rating was calculated as a percentage and was categorized into three groups including poor quality (0%-50%), fair quality (50%-75%), or good quality (75%-100%). Any disagreement in the assessment process was solved through a discussion to reach a consensus.

## Results

**Figure 1:**
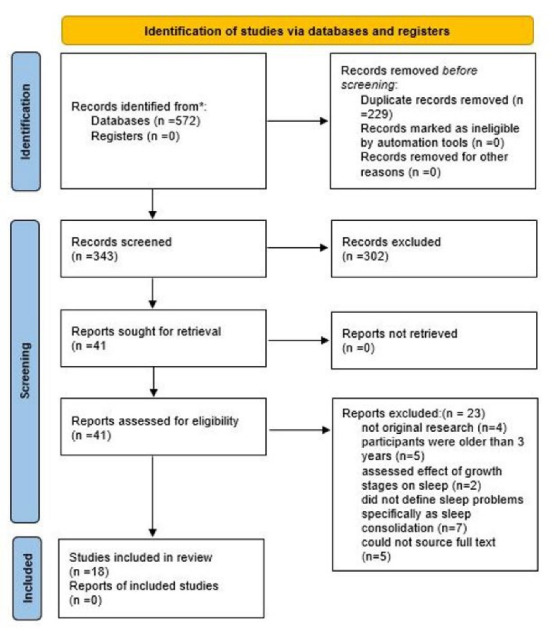
PRISMA flow diagram of included studies

 A total of 572 studies were identified and after removing duplicates, 342 studies remained. Finally, after two-step screening, 18 studies including 16 cohorts and 2 cross-sectional studies met the eligibility criteria (Figure 1). Table S2 provides an overview of the included studies. A total of 10 068 infants/toddlers were assessed at an age range between birth and 36 months. We classified the studies based on their focus on either developmental outcomes or growth indices. Then, we consider the developmental domain(s) (i.e. cognitive, social-emotional, language, and motor development) to sub-categorize them (Table S2).

###  Sleep consolidation and cognitive development

 Eight studies investigated the associations between different aspects of cognitive development and sleep consolidation.^14,15,22-27^ A wide spectrum of outcomes was considered in previous studies including the executive function (EF) and working memory, intelligence quotient (IQ), and mental development index (MDI). Among the studies that assessed the potential associations between EF and night awakenings, two of them found no significant relationships,^14,22^ while Pisch et al reported significant correlations between the number of nights waking as well as wake duration after sleep onset at 4 months and working memory at 6 months. However, it should be noted that these correlations did not replicate in later follow-ups at 8 and 10 months.^24^ In a cohort study among 194 children, night wakings at 6 months were associated with some IQ subscales at 3 years.^25^ Among three studies that used Bayley Scales of Infants and Toddlers Development (BSID) indicated inconsistent results^15,23,26^; two showed no significant association between night wakings and the MDI (as an indicator of cognitive and language development),^15,23^ while the other indicated that more frequent night awakenings (i.e 2 for infants and ≥ 3 for toddlers) were significantly associated with higher and lower MDI, respectively.^26^ Another study that used the cognitive subscale of the Developmental Profile TM-3 questionnaire also found no significant correlation between the number and duration of the night awakening and cognition in 4, and 8-month infants^27^ (Table S2).

###  Sleep Consolidation and Social-Emotional Development

 Seven studies examined relationships between different aspects of social-emotional development and sleep consolidation.^13,27-32^ The tools which were utilized for the assessment of the social-emotional development domain were remarkably varied including Child Behavior Checklist (CBCL),^28^ Ages and Stages Questionnaire: Social-Emotional (ASQ: SE),^13^ Infant Toddler Social Emotional Assessment (ITSEA),^29^Brief Infant-Toddler Social and Emotional Assessment (BITSEA),^30,32^ Interviewer-Completed Computerized Questionnaire (ICCQ)^31^ and Developmental Profile TM-3.^27^ Hall et al,^28^ Mindell et al,^29^ and Pecora et al^27^ found no significant association between uninterrupted sleep and internalizing and/or externalizing behaviors in children. On the other hand, Morales-Muñoz et al^30^ and Zaidman-Zait & Hall^31^ and Mäkelä et al^32^ reported some significant relationships. Moreover, in a large cross-sectional study among 2041 two-year children, significant associations between ≥ 3 nocturnal awakenings as well as > 30 minutes night awakenings and social-emotional problems were observed.^13^

###  Sleep consolidation and language development

 To the best of our knowledge, five studies assessed the association between sleep consolidation and language development.^12,15,23,26,27^ Among the included studies, only two studies reported some significant relationships at some specific time points.^12,27^ Dionne et al indicated that children with more consolidated sleep at 18 months had better language skills at 5 years of age.^12^ Pecora et al also found a negative correlation between the number of night waking at 4, and 8 months with language development at 8 months.^27^ In another study among 590 infants and 512 toddlers, higher rates of night awakenings (i.e 2 for infants and ≥ 3 for toddlers) were significantly associated with higher and lower MDI (an indicator of the level of children’s cognitive and language development), respectively.^26^ In contrast, two other studies found no significant association between uninterrupted nighttime sleep and language developmental outcomes.^15,23^ (Table S2)

###  Sleep consolidation and fine and gross motor development

 We finally included three studies assessing the relationships between sleep consolidation and motor development.^15,23,26^ Overall, findings among 1641 children indicated that early-onset fragmented sleep did not have either a negative or positive effect on motor development within the first years of life. All three studies were consistent regarding the methods which were used for sleep and motor development assessments and none of them utilized the actigraphy for night waking assessment (Table S2).

###  Sleep consolidation and growth

 To our knowledge, three studies investigated the potential association between sleep consolidation and growth.^16,33,34^ In general, no studies revealed significant associations between sleep consolidation and anthropometric indices in children. Petrov et al followed up with 126 infants between 1 to 36 months of age and found no significant association between their longest nocturnal sleep bout and obesity outcomes at 6 and 36 months of age. Outcome measures included rapid weight gain (RWG) at 6 months of age which was defined as > 0.67 positive change in weight for age Z-score from birth to 6 months and incident overweight (OW) at 36 months which was defined as BMI percentile ≥ 85 using the Centers for Disease Control and Prevention growth charts.^33^ Similarly, in another large cohort study, Wang et al. showed no significant association between night awakenings > 3 and child BMI z score at 6, 14, and 36 months of age.^16^ These results were consistent with Tikotzky and colleagues’ findings using both questionnaire and actigraphy, a more reliable method for night awakenings evaluation. In their studies, they assessed the relationship between the number of night wakings and four anthropometric indices including weight, height, weight for length ratio (WLR), and weight above weight expected for length (WEFL)^34^ in 6-month infants (Table S2).

###  Quality assessment 

 Based on the NIH’s quality assessment tool for observational studies, eleven and seven studies had good and fair quality, respectively. Table S3 provides an overview of the quality scores and status.

## Discussion

 This systematic review synthesized peer-reviewed scientific evidence from 18 studies examining the relationships between sleep consolidation and growth and development in early childhood (0-3 years).

 The fact that we get large amounts of sleep in the early years of life suggests that sleep may play a role in brain development and developmental plasticity. This idea has been explored through various studies in animals and humans, but evidence remains unclear.^35-37^ Sleep plays a crucial role in different stages of growth and development in humans. Based on the existing evidence, the body releases GHs during sleep that can promote physical growth and facilitate repairing tissues.^38^ Sleep also plays a critical role in the regulation of appetite and metabolism, which can affect weight gain and obesity risk in children.^39^ In addition, sleep enhances cognitive processes such as memory consolidation, learning, and academic performance.^40^ It has been shown that sleep disturbances such as frequent night waking can lead to daytime sleepiness and irritability, which can affect behavior and emotional regulation in children and adolescents.^41,42^ Therefore, we hypothesized that disruptions in sleep patterns can have negative effects on both physical growth and development in children with a particular focus on early life. Overall, the effects of sleep on child growth and development are multifaceted and involve complex physiological processes. Therefore, it is important to prioritize healthy sleep habits in children to promote optimal growth and development. Parenting education and intervention programs in this regard can improve growth and development in children.^42^

 In total, maintenance of sleep consolidation is a common controversial problem in practice in infants and toddlers, that has various effects on mothers and children. One of the main problems of research on sleep consolidation is that there is not a unified definition among researchers and studies, therefore studies used various variables and methods to measure sleep consolidation in early childhood. Sleep consolidation generally is defined as uninterrupted sleep through the night based on age. Thus, we reviewed all studies that measured this concept via either duration of uninterrupted sleep through night or night wakening index to evaluate their impact on child growth and development. As it was predictable, the results were not consistent. Hence, this makes us unable to conclude the effect of sleep consolidation on child growth and development strongly. We highly recommend researchers define sleep maturation precisely for different ages to lower this heterogeneity across studies.

 Overall, in this review, the potential associations between sleep consolidation and child development particularly cognitive, social-emotional, and language were noted. However, no studies supported the association between sleep consolidation and motor development or growth indices.

 Previous studies showed that sleep deprivation can develop less connectivity between the prefrontal cortex and amygdala which has a negative effect on social-emotional development.^21,43^ Additionally, it can also increase oxidative stress markers in the cortex, amygdala, and hippocampus, which can affect the regulation of sleep deprivation-related behavioral impairments.^44^

 In this study, we found that frequent night awakenings can affect some aspects of development. The same methods of collecting sleep and development information should be utilized in future studies to let us have more interpretable results. It is better to use objective measurement for both sleep and development. In total, the included studies in this field reported conflicting results regarding the association between sleep consolidation and child growth and development. The potential reasons that can explain this heterogeneity include (1) differences in sociodemographic characteristics such as parental characteristics, which affect the interpretation of parents regarding the definition of sleep consolidation (the number and duration of night awakenings), (2) the different cut-offs of night wakings and duration of uninterrupted sleep through the night across different ages, (3) differences in sleep-related covariate factors including sleep habits such as co-sleeping, night feeding, use of self-soothing methods to fall asleep and sleep duration, (4) differences in study design and sample size, (5) variety in age range and developmental stage in different studies, and (6) utilizing different tools to assess sleep (e.g. sleep diary, ISQ, BISQ, actigraphy, etc.) and development (e.g. BSID, ASQ, CBCL, etc.) in infants and toddlers.

## Strengths and limitations

 To our knowledge, this is the most recent comprehensive systematic review on the association between sleep consolidation and growth or development in early childhood. As we explored the data from various studies, we encountered significant heterogeneity in the methodologies, populations, and outcome measures reported. This variability posed considerable challenges to conducting a robust meta-analysis and quantitative approach. Given these differences, aggregating the data in a way that could misrepresent the findings or lead to misleading conclusions was not appropriate. Instead, we focused on systematically classifying the outcomes (cognitive development, social-emotional development, language development and motor development as well as growth indices) as clearly as possible. Our intention was to provide a comprehensive overview that highlights the existing evidence while also illustrating the diversity of results across studies. By presenting this classification in Table S2, we aimed to provide a clearer picture of the current landscape, enabling the researchers to identify trends, strengths, and weaknesses in the existing literature.

 While this systematic review provides valuable insights in this field, several limitations must be acknowledged. Firstly, the heterogeneity among the included studies in terms of study design, sample size, and measurement tools for assessing sleep consolidation and developmental outcomes poses a significant challenge. The varying definitions and methods used to measure sleep patterns, such as different criteria for what constitutes “night wakings” or “consolidated sleep,” could lead to inconsistencies in the findings and limit the comparability across studies. Secondly, the observational nature of the included studies precludes the establishment of causal relationships. While associations between sleep consolidation and developmental outcomes were observed, it is not possible to definitively conclude that sleep patterns directly influence growth or development, as other unmeasured confounding variables, such as genetic factors, parenting practices, and socioeconomic status, may also play significant roles. Thirdly, the reliance on parental reporting for sleep patterns and developmental milestones in many studies introduces the potential for bias. Parents may inaccurately recall or report their child’s sleep behaviors, leading to misclassification or measurement errors that could affect the study outcomes. Furthermore, cultural differences in sleep practices and parental expectations were not consistently accounted for, which might influence the generalizability of the findings across different populations. Moreover, the quality of the included studies varied, with some studies being rated as fair or poor quality based on the NIH quality assessment tool. The methodological limitations, such as small sample sizes, short follow-up periods, and lack of control for potential confounders in some studies, may have impacted the robustness of the results. Lastly, the review was restricted to studies published in English, which may have resulted in language bias and the exclusion of relevant studies published in other languages. Additionally, publication bias cannot be ruled out, as studies with significant findings are more likely to be published, potentially skewing the overall conclusions of this review.

 Future research should aim to address these limitations by conducting more rigorous, large-scale, longitudinal studies with standardized measures of sleep and developmental outcomes, and by including diverse populations to enhance the generalizability of the findings.

## Conclusion

 To the best of our knowledge, this is the first systematic review assessing the effect of sleep consolidation on growth and development in early childhood (from birth to 3 years of age). According to the existing evidence, at least, the potential associations between sleep consolidation and child development particularly cognitive, social-emotional, and language development cannot be ruled out. However, due to the heterogenicity of results and inconsistent findings in some studies, we cannot still strongly declare that sleep consolidation is a remarkable predictor for child development. On the other hand, no studies supported the association between sleep consolidation and motor development or growth indices. Future longitudinal studies should consider a combination of more objective (actigraphy) and subjective measures (sleep logs and questionnaires) for measuring sleep, in addition to objective standard tools for evaluating different domains of development. Investigating night waking cut-offs across different ages in early childhood in future studies would provide valuable information to establish practical guidelines for infant and toddler sleep.

## Competing Interests

 The authors declare no conflict of interest.

## Ethical Approval

 Not applicable.

## Supplementary Files


Supplementary file 1 contains Table S1, S2 and S3.

## References

[1] Burnham MM, Goodlin-Jones BL, Gaylor EE, Anders TF (2002). Nighttime sleep-wake patterns and self-soothing from birth to one year of age: a longitudinal intervention study. J Child Psychol Psychiatry.

[2] Goodlin-Jones BL, Burnham MM, Gaylor EE, Anders TF (2001). Night waking, sleep-wake organization, and self-soothing in the first year of life. J Dev Behav Pediatr.

[3] Owens JA, Mindell JA (2011). Pediatric insomnia. Pediatr Clin North Am.

[4] Yates J (2018). PERSPECTIVE: the long-term effects of light exposure on establishment of newborn circadian rhythm. J Clin Sleep Med.

[5] Schechtman VL, Harper RK, Harper RM (1994). Distribution of slow-wave EEG activity across the night in developing infants. Sleep.

[6] Henderson JM, France KG, Owens JL, Blampied NM (2010). Sleeping through the night: the consolidation of self-regulated sleep across the first year of life. Pediatrics.

[7] Teng A, Bartle A, Sadeh A, Mindell J (2012). Infant and toddler sleep in Australia and New Zealand. J Paediatr Child Health.

[8] Baddam S, Nasser A (2021). A clinical guide to pediatric sleep: diagnosis and management of sleep problems, third edition. J Am Acad Child Adolesc Psychiatry.

[9] Zaffanello M, Pietrobelli A, Cavarzere P, Guzzo A, Antoniazzi F (2023). Complex relationship between growth hormone and sleep in children: insights, discrepancies, and implications. Front Endocrinol (Lausanne).

[10] Paller KA, Creery JD, Schechtman E (2021). Memory and sleep: how sleep cognition can change the waking mind for the better. Annu Rev Psychol.

[11] Yazdi M, Bemanalizadeh M, Kelishadi R (2024). Persian version of brief infant sleep questionnaire (BISQ): a psychometric evaluation. BMC Pediatr.

[12] Dionne G, Touchette E, Forget-Dubois N, Petit D, Tremblay RE, Montplaisir JY (2011). Associations between sleep-wake consolidation and language development in early childhood: a longitudinal twin study. Sleep.

[13] Hysing M, Sivertsen B, Garthus-Niegel S, Eberhard-Gran M (2016). Pediatric sleep problems and social-emotional problems. A population-based study. Infant Behav Dev.

[14] Mäkelä TE, Peltola MJ, Saarenpää-Heikkilä O, Himanen SL, Paunio T, Paavonen EJ (2020). Night awakening and its association with executive functioning across the first two years of life. Child Dev.

[15] Pennestri MH, Laganière C, Bouvette-Turcot AA, Pokhvisneva I, Steiner M, Meaney MJ (2018). Uninterrupted infant sleep, development, and maternal mood. Pediatrics.

[16] Wang L, Jansen W, Boere-Boonekamp MM, Vlasblom E, L’Hoir MP, Beltman M (2019). Sleep and body mass index in infancy and early childhood (6-36 mo): a longitudinal study. Pediatr Obes.

[17] Bathory E, Tomopoulos S (2017). Sleep regulation, physiology and development, sleep duration and patterns, and sleep hygiene in infants, toddlers, and preschool-age children. Curr Probl Pediatr Adolesc Health Care.

[18] Liberati A, Altman DG, Tetzlaff J, Mulrow C, Gøtzsche PC, Ioannidis JP (2009). The PRISMA statement for reporting systematic reviews and meta-analyses of studies that evaluate health care interventions: explanation and elaboration. J Clin Epidemiol.

[19] Page MJ, McKenzie JE, Bossuyt PM, Boutron I, Hoffmann TC, Mulrow CD (2021). Updating guidance for reporting systematic reviews: development of the PRISMA 2020 statement. J Clin Epidemiol.

[20] von Elm E, Altman DG, Egger M, Pocock SJ, Gøtzsche PC, Vandenbroucke JP (2008). The Strengthening the Reporting of Observational Studies in Epidemiology (STROBE) statement: guidelines for reporting observational studies. J Clin Epidemiol.

[21] Foley JE, Weinraub M (2017). Sleep, affect, and social competence from preschool to preadolescence: distinct pathways to emotional and social adjustment for boys and for girls. Front Psychol.

[22] Bernier A, Carlson SM, Bordeleau S, Carrier J (2010). Relations between physiological and cognitive regulatory systems: infant sleep regulation and subsequent executive functioning. Child Dev.

[23] Mäkelä TE, Peltola MJ, Nieminen P, Paavonen EJ, Saarenpää-Heikkilä O, Paunio T (2018). Night awakening in infancy: developmental stability and longitudinal associations with psychomotor development. Dev Psychol.

[24] Pisch M, Wiesemann F, Karmiloff-Smith A (2019). Infant wake after sleep onset serves as a marker for different trajectories in cognitive development. J Child Psychol Psychiatry.

[25] Plancoulaine S, Stagnara C, Flori S, Bat-Pitault F, Lin JS, Patural H (2017). Early features associated with the neurocognitive development at 36 months of age: the AuBE study. Sleep Med.

[26] Sun W, Li SX, Jiang Y, Xu X, Spruyt K, Zhu Q (2018). A community-based study of sleep and cognitive development in infants and toddlers. J Clin Sleep Med.

[27] Pecora G, Focaroli V, Paoletti M, Barca L, Chiarotti F, Borghi AM (2022). Infant sleep and development: concurrent and longitudinal relations during the first 8 months of life. Infant Behav Dev.

[28] Hall WA, Scher A, Zaidman-Zait A, Espezel H, Warnock F (2012). A community-based study of sleep and behaviour problems in 12- to 36-month-old children. Child Care Health Dev.

[29] Mindell JA, Leichman ES, DuMond C, Sadeh A (2017). Sleep and social-emotional development in infants and toddlers. J Clin Child Adolesc Psychol.

[30] Morales-Muñoz I, Lemola S, Saarenpää-Heikkilä O, Kylliäinen A, Pölkki P, Paunio T (2020). Parent-reported early sleep problems and internalising, externalising and dysregulation symptoms in toddlers. BMJ Paediatr Open.

[31] Zaidman-Zait A, Hall WA (2015). Children’s night waking among toddlers: relationships with mothers’ and fathers’ parenting approaches and children’s behavioural difficulties. J Adv Nurs.

[32] Mäkelä TE, Kylliäinen A, Saarenpää-Heikkilä O, Paavonen EJ, Paunio T, Leppänen JM (2021). Signaled night awakening and its association with social information processing and socio-emotional development across the first two years. Sleep.

[33] Petrov ME, Whisner CM, McCormick D, Todd M, Reyna L, Reifsnider E (2021). Sleep-wake patterns in newborns are associated with infant rapid weight gain and incident adiposity in toddlerhood. Pediatr Obes.

[34] Tikotzky L, G DEM, Har-Toov J, Dollberg S, Bar-Haim Y, Sadeh A (2010). Sleep and physical growth in infants during the first 6 months. J Sleep Res.

[35] Peirano PD, Algarín CR (2007). Sleep in brain development. Biol Res.

[36] Frank MG (2011). Sleep and developmental plasticity not just for kids. Prog Brain Res.

[37] Graven S (2006). Sleep and brain development. Clin Perinatol.

[38] Rose M, Sanford A, Thomas C, Opp MR (2001). Factors altering the sleep of burned children. Sleep.

[39] Knutson KL, Spiegel K, Penev P, Van Cauter E (2007). The metabolic consequences of sleep deprivation. Sleep Med Rev.

[40] Capellini I, McNamara P, Preston BT, Nunn CL, Barton RA (2009). Does sleep play a role in memory consolidation? A comparative test. PLoS One.

[41] Sadeh A (2007). Consequences of sleep loss or sleep disruption in children. Sleep Med Clin.

[42] Heidari-Beni M, Bemanalizadeh M, Heshmat R, Qorbani M, Kelishadi R (2022). Changes in lifestyle behaviors of children and adolescents during the COVID-19 pandemic and the impact on the development of non-communicable diseases: a narrative review. Med J Islam Repub Iran.

[43] Havekes R, Vecsey CG, Abel T (2012). The impact of sleep deprivation on neuronal and glial signaling pathways important for memory and synaptic plasticity. Cell Signal.

[44] Alrousan G, Hassan A, Pillai AA, Atrooz F, Salim S (2022). Early life sleep deprivation and brain development: insights from human and animal studies. Front Neurosci.

