# Supplementary material for: The association between sleep consolidation and growth and development in early childhood: A systematic review
Source: Health Promot Perspect. 2024 Dec 30;14(4):312–8. doi: 10.34172/hpp.43037 (PMC11873771; doi:10.34172/hpp.43037)
Supplement: Supplementary file 1 — contains Table S1, S2 and S3. [file hpp-14-312-s001.pdf]

Bemanalizadeh et al, **Health Promotion Perspectives.** 2024;14(4):312-S1
doi: 10.34172/hpp.43037
https://hpp.tbzmed.ac.ir

Supplementary file 1

Table S1- Keywords and alternative words used in the search strategy

**Keywords**	**Synonyms (Alternative keywords)**
Growth	body weight<br>body height<br>body size
Development	child development<br>infant development
Sleep consolidation	night feeding<br>night-time waking<br>night awakening<br>infant sleep

Table S2.Description of the included studies

Number	Author, publication year	Study design, country	Sample size	Age	Sleep parameters	Sleep Assessment	Outcome	Outcome assessment	Covariates	Main findings
**Cognitive development**										
1	Bernier[22] 2010	Cohort study, Canada	60	12-26 months	Number of night awakenings (fragmentation) (at least 30 min of consecutive sleep)	Parent sleep diary at 12 and 18 months	EF (working memory, conflict-EF and impulse control) at 18 and 26 months	EF at 18 months: *Hide the pots* to assess working memory<br><br>EF at 26 months: *Spin the Pots, Shape Stroop, Baby Stroop and Delay of Gratification* to assess working memory, inhibitory control, and set shifting, delay or impulse control tasks	-	12-month sleep fragmentation and 18-month working memory: correlation coefficient: 0.03, p>0.05<br><br>12-month sleep fragmentation and 26-month conflict-EF: correlation coefficient: -0.03, p>0.05<br><br>12-month sleep fragmentation and 26-month impulse control: correlation coefficient: 0.04, p>0.05<br><br>18-month sleep fragmentation and 18-month working memory: correlation coefficient: 0.12, p>0.05<br><br>18-month sleep fragmentation and 26-

										month conflict-EF: correlation coefficient: -0.07, p>0.05<br><br>18-month sleep fragmentation and 26-month impulse control: correlation coefficient: 0.01, p>0.05
2	Mäkelä[14] 2020	Cohort study, Finland	145	8 -24 months	Number of night awakenings (between midnight and 6 a.m)<br><br>waking group (night waking ≥ 3 awakenings) and nonwaking group (≤ 1 awakening)	Parent-reported sleep log and actigraphy for three consecutive days at 8 months	EF at 8 and 24 months	EF at 8 months: *Switch task* to assess the ability to learn new stimulus sequences and to learn a new conflicting response<br><br>EF at 24 months: *Switch task*, *Spin the Pots* to assess working memory, *and Snack Delay task* to assess inhibitory control, *BRIEF-P* to assess EF (*ISCI*, inhibit and emotional control scales, *FI:* shift and working memory, and *EMC*: working memory and plan/organize)	Co-sleeping, breastfeeding, ability to fall asleep alone, healthcare center status, sleep duration, and time spent awake during the night	The overall performance of the waking and nonwaking group in the *Switch task*: p >0.05<br><br>The performance of the waking (M = 7.53, SD = 2.612) and nonwaking group (M = 7.33, SD = 2.678) in the *Spin the Pot task*: t (116) = 0.405, p = 0.686<br><br>The performance of the waking (M = 36.7 s, SD = 24.0 s) and nonwaking group (M = 39.3 s, SD = 23.3 s) in the *Snack Delay task*: t (116) = -0.577, p = 0.565

										The performance of the waking and nonwaking group in any of the different *BRIEF-P indexes*: p>0.05, e.g. the performance of the waking (M = 50.13, SD = 9.10) and nonwaking group (M = 50.03, SD = 8.92) in *ISCI* index of *BRIEF-P*: t (83) = 0.05, p = 0.961; the performance of the waking (M = 64.33, SD = 11.97) and nonwaking group (M = 60.38, SD = 9.17) in *FI* index of *BRIEF-P*: t (83) = 1.668, p = 0.099; the performance of the waking (M = 55.23, SD = 12.98) and nonwaking group (M = 51.73, SD = 10.35) in *EMI* index of *BRIEF-P*: t(83) = 1.343, p = 0.183)
3	Pisch [24] 2019	Cohort study, Germany	40	4-10 months	Night waking frequency, and duration of wake after sleep onset	Parent sleep diary, BISQ, and actigraphy for seven consecutive nights at 4,6,8,10 months	EF	A working memory task based on visual and auditory stimuluses at 6,8,10 months	-	Correlation between duration of wake after sleep onset at 4 months and memory at 6 months: 0.41, p <0.05

										Correlation between duration of wake after sleep onset at 4 months and memory at 8 months: 0.32, p >0.05<br><br>Correlation between duration of wake after sleep onset at 4 months and memory at 10 months: 0.22, p >0.05<br><br>Correlation between duration of wake after sleep onset at 6 months and memory at 8 months: 0.17, p >0.05<br><br>Correlation between duration of wake after sleep onset at 6 months and memory at 10 months: 0.26, p >0.05<br><br>Correlation between duration of wake after sleep onset at 8 months and memory at 10 months: 0.24, p >0.05

										Correlation between duration of wake after sleep onset at 8 months and memory at 6 months: 0.53, $p < 0.01$<br><br>Correlation between night waking frequency at 4 months and memory at 8 months: 0.4, $p > 0.05$<br><br>Correlation between night waking frequency at 4 months and memory at 10 months: 0.17, $p > 0.05$<br><br>Correlation between night waking frequency at 6 months and memory at 8 months: 0.38, $p > 0.05$<br><br>Correlation between night waking frequency at 6 months and memory at 10 months: 0.37, $p > 0.05$<br><br>Correlation between night waking frequency at 8 months and memory at 10

										Correlation between night waking frequency at 4 months and months: 0.28, p >0.05
4	Plancoulaine[25] 2017	Cohort study, France	194	6-36 months	Night awakenings	BISQ at 6, 12, 18, and 24 months	IQ (IQ)	*Weschler Preschool and Primary Scale Intelligence−III* (Receptive Vocabulary, Block Design, Information, Object Assembly, and Picture Naming to assess VIQ, PIQ and FSIQ) at 36 months	Sociodemographic characteristics (Maternal work category), Maternal characteristics (Pre-pregnancy BMI, smoking during pregnancy) and Child characteristics (Term at birth, TV watching at age 2 yr, Birth rank)	Night awakenings at 6 months and *VIQ* at 36 months: unadjusted: β= -2.44, SD= 2.08, p>0.05; adjusted: β= -2.06, SD=1.98, p>0.05<br><br>Night awakenings at 6 months and *PIQ* at 36 months: unadjusted: β= -4.34, SD= 1.79, p<0.05; adjusted: β= -3.89, SD= 1.65, p<0.05<br><br>Night awakenings at 6 months and *FSIQ* at 36 months: β= -3.81, SD=1.87, p<0.05; adjusted: β= -3.34, SD= 1.69, p<0.05<br><br>Night awakenings at 12 months and *VIQ* at 36 months: β= -1.57, SD= 2.07, p>0.05<br><br>Night awakenings at 12 months and *PIQ* at 36 months: β= 0.89, SD= 1.80, p>0.05

										Night awakenings at 12 months and *FSIQ* at 36 months: β= -0.46, SD=1.88, p>0.05<br><br>Night awakenings at 18 months and *VIQ* at 36 months: β= 0.32, SD= 2.10, p>0.05<br><br>Night awakenings at 18 months and *PIQ* at 36 months: β= -0.75, SD= 1.83, p>0.05<br><br>Night awakenings at 18 months and *FSIQ* at 36 months: β= -0.2, SD=1.90, p>0.05<br><br>Night awakenings at 24 months and *VIQ* at 36 months: β= -1.36, SD= 2.08, p>0.05<br><br>Night awakenings at 24 months and *PIQ* at 36 months: β= -3.55, SD= 1.79, p>0.05<br><br>Night awakenings at 24 months and *FSIQ* at 36 months: β= -2.87 SD=1.87, p>0.05

5	Mäkelä[23] 2018	Cohort study, Finland	151	8-24 months	Number of night awakenings (between midnight and 6 a.m)<br><br>waking group (night waking ≥ 3 awakenings) and nonwaking group (≤ 1 awakening)	BISQ at 8 months	Cognitive subscale	BSID-III at 8 and 24 months	-	No significant main effect in cognitive subscale between waking an nonwaking groups:<br>Estimate (SE) = 0.09 (0.23), t= 0.40, p= 0.689
6	Pennestri[15] 2018	Cohort study, Canada	At 6 months : 388, at 12 months : 369	6-12 months	6 or 8 hours of uninterrupted sleep through the night	Self-administered questionnaire asking about the infants' sleep habits at 6 and 12 months	Mental index including cognitive and language development level	BSID-II at 6 and 12 months	Infant's sex, socioeconomic status, breastfeeding status, co-sleeping status, and total sleep duration in a 24-hour period	Association between sleeping through the night or not at 6 and 12 months using the 6-hour criterion and mental development:<br>At 6 months:<br>$\beta = -1.78$, $p = 0.10$<br>At 12 months:<br>$\beta = -1.19$, $p = 0.45$<br><br>Association between sleeping through the night or not at 6 and 12 months using the 8-hour criterion and mental development:<br>At 6 months:<br>$\beta = -1.53$, $p = 0.15$<br>at 12 months:<br>$\beta = -0.92$, $p = 0.50$

7	Sun[26] 2018	Cross-sectional study, China	Infant: 590, Toddler: 512	2-30 months	Frequency of night awakenings	BISQ	MDI including cognitive and language development level	BSID-I	Recruited province, child's age and sex, birth weight, maternal education level, bedroom sharing and being currently breastfed	Association between sleep infants' nighttime awakenings and MDI:<br>1×/night:<br>β±SE: 1.45 ± 2.56, p= 0.57<br>2×/night:<br>β±SE: 6.03 ± 2.43 p= 0.01<br>≥ 3×/night:<br>β±SE: 2.30 ± 2.38, p=0.34<br><br>Association between sleep toddlers' nighttime awakenings and MDI:<br>1×/night<br>β±SE: −0.09 ± 1.79, p= 0.96<br>2×/night<br>β±SE: 0.20 ± 1.99, p= 0.92<br>≥ 3×/night<br>β±SE: −5.15 ± 2.39, p= 0.03
8	Pecora[27] 2022	Cohort study, Italy	156	4-8month	Night awakening number and duration	BISQ	Cognitive subscale	Developmental ProfileTM-3	Breastfeeding, using a pacifier, siblings, co-sleeping	Correlation between number of night waking and cognitive subscale at 4month:-.02, p>.05<br><br>Correlation between duration of night waking and cognitive

										subscale at 4month:.07, p>.05<br><br>Correlation between number of the night waking at 4month and cognitive subscale at 8month: -.02, p>.05<br><br>Correlation between duration of the night waking at 4month and cognitive subscale at 8month:0.06, p>.05<br><br>Correlation between number of the night waking and cognitive subscale at 8month:0.4, p>.05<br><br>Correlation between duration of night waking and cognitive subscale at 8 month: -.0.4, p>.05
**Social-emotional development**										
1	Hall[28] 2012	Cross-sectional study, Canada	58	12-36 months	Night waking index: the number of	ISQ	Behavioral problems	CBCL	-	Correlation coefficient between night waking index and internalizing behavior: 0.13, p>0.0

					interrupted nights, the number of awakenings per night, the average time spent awake, and the duration of waking difficulties the number of interrupted nights, the number of awakenings per night, and the average time spent awake, and the duration of waking difficulties					5<br><br>Correlation coefficient between night waking index and internalizing behavior: 0.11, p>0.05<br><br>Correlation coefficient between night waking index and total score of CBCL: 0.25, p>0.05
2	Hysing[13] 2016	Cohort study, Norway	2041	24 months	Wake after sleep onset at night (more or less than 30 minutes), number of night awakenings	BISQ at 24 months	Social and emotional development	ASQ:SE at 24 months	Maternal age, maternal education, marital status, parity, gestation, child sex, and child birth weight and three subscales of ASQ questionnaire	Association between number of nocturnal awakenings and social-emotional problems:<br>fully adjusted OR (95%CI) for 1-2 awakenings: 1.72 (0.7-4.06)<br>fully adjusted OR (95%CI) for ≥3 awakenings: 1.95 (1.16-3.27)

									(Communication problems, Gross motor problems, and Fine motor problems)	Association between duration of wake after sleep onset and social-emotional problems: fully adjusted OR (95%CI)<br>for >30 min: 3.25 (1.80-5.86)
3	Mindell[29] 2017	Cohort study, United states	117	6-18 months	Longest continuous sleep period at night and number of the night awakenings	BISQ at 6, 12, and 18 months	Social-emotional development (externalizing and internalizing problems)	ITSEA at 12 and 18 months	-	Correlation coefficient between number of night wakings and longest sleep period at 6 months and externalizing behavior at 12 months: -0.46, p >0.05, and 0.63, p >0.05<br><br>Correlation coefficient between number of night wakings and longest sleep period at 6 months and externalizing behavior at 18 months: 0.056, p >0.05, and 0.252, p>0.05<br><br>Correlation coefficient between number of night wakings and longest sleep period at 12 months and externalizing behavior at 12 months: -0.053,

										p >0.05, and 0.025, p>0.05<br><br>Correlation coefficient between number of night wakings and longest sleep period at 12 months and externalizing behavior at 18 months: -0.040, p >0.05, and 0.057, p>0.05<br><br>Correlation coefficient between number of night wakings and longest sleep period at 18 months and externalizing behavior at 12 months: -0.030, p >0.05, and 0.012, p>0.05<br><br>Correlation coefficient between number of night wakings and longest sleep period at 18 months and externalizing behavior at 18 months: -0.117, p >0.05, and 0.065, p>0.05<br><br>Correlation coefficient between number of night wakings and

										longest sleep period at 6 months and internalizing behavior at 12 months: 0.196, p >0.05, and -0.116, p>0.05<br><br>Correlation coefficient between number of night wakings and longest sleep period at 6 months and internalizing behavior at 18 months: -0.032, p >0.05, and 0.207, p>0.05<br><br>Correlation coefficient between number of night wakings and longest sleep period at 12 months and internalizing behavior at 12 months: -0.018, p >0.05, and 0.010, p>0.05<br><br>Correlation coefficient between number of night wakings and longest sleep period at 12 months and internalizing behavior at 18 months: 0.135, p >0.05, and -0.218, p>0.05

										Correlation coefficient between number of night wakings and longest sleep period at 18 months and internalizing behavior at 12 months: -0.017, p >0.05, and 0.074, p>0.05<br><br>Correlation coefficient between number of night wakings and longest sleep period at 18 months and internalizing behavior at 18 months: 0.065, p >0.05, -0.49, p>0.05
4	Morales-Munoz[30] 2020	Cohort Study, Finland	936	3-24 months	Number of night awakenings, Proportion of day time sleep per total sleep	BISQ and ISQ at 3, 8, 18 and 24 months	Social emotional development (externalizing and internalizing problems)	BITSEA at 24 months	Child's age, child's sex, maternal age, maternal education, maternal health and gestational age	Associations between number of night wakings at 3 months and externalizing symptoms at 24 months: β (95%CI) = 0. 10 (0.05-0.29), p= 0.006<br><br>Associations between number of nigh wakings at 8 months and externalizing symptoms at 24 months: β(95%CI) = 0.02 (0.08- 0.15), p= 0.534

										Associations between number of nigh wakings at 18 months and externalizing symptoms at 24 months: β(95%CI) = 0.07 (0.00- 0.26), p= 0.057<br><br>Associations between number of nigh wakings at 24 months and externalizing symptoms at 24 months: β(95%CI) = 0.07 (0.01- 0.31), p= 0.047<br><br>Associations between number of nigh wakings at 8 months and internalizing symptoms at 24 months: β=0.07 (0.00- 0.15), p= 0.049<br><br>Associations between number of nigh wakings at 18 months and internalizing symptoms at 24 months: β=0.08 (0.02- 0.20), p= 0.019

										Associations between number of nigh wakings at 24 months and internalizing symptoms at 24 months: β=0.11 (0.06-0.27), p= 0.002
5	Zaidman-Zait,[31] 2015	Cohort study, Canada	1487	5-29 months	Night waking length	SAQM at 29 months	Child behavior /symptoms (Aggression, hyperactivity, opposition, shyness/inhibition anxiety & depression separation anxiety)	ICCQ at 29 months	-	Significant main effects for waking at night (>20 minutes) for all externalizing (aggression(F=4.61), hyperactivity(F=10.34) and opposition(F=5.85)) and internalizing behaviors (Shyness/inhibition(F= 4.21), Anxiety & depression(F=2.66) and Separation anxiety(F=20.02)) at 29 months
6	Mäkelä[32] 2021	Cohort study, Finland	146	8-24 month	Number of night awakenings: waking group (night waking ≥ 3 awakenings) and nonwaking group (≤ 1 awakening)	BISQ and actigraphy at 8,24month	Social emotional development (internalizing, externalizing , dysregulation and social competence)	BITSEA at 24 months	The amount of breastfeeding , co-sleeping, infant's ability to fall asleep alone	Mean (SD) differences between waking and nonwaking groups in dysregulation: 4.23± 2.74, 2.47± 2.14, p=0.002<br><br>Mean (SD) differences between waking and nonwaking groups in social competence:

										18.11± 2.42, 19.13± 2.60, p=0.049<br><br>Mean (SD) differences between waking and nonwaking groups in externalizing: 3.41± 2.27, 3.00± 2.10, p=0.355<br><br>Mean (SD) differences between waking and nonwaking groups in internalizing: 1.59± 1.75, 1.05± 0.99, p=0.065<br><br>Mean (SD) differences between waking and nonwaking groups in dysregulation(no sleep): 2.42± 1.81, 1.79± 1.53, p=0.049
7	Pecora[27] 2022	Cohort study, Italy	156	4-8month	Night awakening number and duration	BISQ	Socio-emotional subscale	Developmental ProfileTM-3	Breast feeding, using pacifier, siblings, co-sleeping	Correlation between number of night waking and Socio-emotional subscale at 4month:-.04 , p>.05<br><br>Correlation between duration of night waking and Socio-

											emotional subscale at 4month:.-0.01 , p>.05<br><br>Correlation between number of the night waking at 4month and Socio-emotional subscale at 8month: -.03, p>.05<br><br>Correlation between duration of the night waking at 4month and Socio-emotional subscale at 8month:0.01, p>.05<br><br>Correlation between number of the night waking and Socio-emotional subscale at 8month:-0.08, p>.05<br><br>Correlation between duration of night waking and Socio-emotional subscale at 8month:-.0.13 , p>.05
**Language development**											
1	Dionne[12] 2011	Cohort study, Canada	1029	6-30 months	Consecutive hours of sleep	Mothers' interview	Language skills	MCDI-SF at 18,30 months	Child sex, birth weight,	Spearman correlation between consecutive

					at night (<4, 5, 6, 7, 8 or >8 at 6 months and <4, 5, 6, 7, 8, 9, 10, or >10 at 18 and 30 months				gestation duration, average 1 and 5 min Apgar score at birth, number of days spent in hospital after birth, and difficult temperament, Maternal education, family income, average number of cigarettes smoked per day throughout pregnancy, maternal depressive symptoms at 6 and 18 months, mother's perceived parental impact, and maternal overprotection	nighttime sleep at 6 months and language outcomes at 18 months:<br>-0.04, p>0.05<br><br>Spearman correlation between consecutive nighttime sleep at 6 months and language outcomes at 30 months: 0.03, p>0.05<br><br>Spearman correlation between consecutive nighttime sleep at 6 months and language outcomes at 60 months: 0.05, p>0.05<br><br>Spearman correlation between consecutive nighttime sleep at 18 months and language outcomes at 18 months:<br>-0.05, p>0.05<br><br>Spearman correlation between consecutive nighttime sleep at 18 months and language outcomes at 30 months: 0.06, p>0.05

										Spearman correlation between consecutive nighttime sleep at 18 months and language outcomes at 60 months: 0.12, p<0.01<br><br>Spearman correlation between consecutive nighttime sleep at 30 months and language outcomes at 18 months:<br>0.05, p>0.05<br><br>Spearman correlation between consecutive nighttime sleep at 30 months and language outcomes at 30 months: 0.05, p>0.05<br><br>Spearman correlation between consecutive nighttime sleep at 30 months and language outcomes at 60 months: 0.05, p>0.05
2	Mäkelä[23] 2018	Cohort study, Finland	151	8-24 months	Number of night awakenings (between midnight and 6 a.m)	BISQ at 8 months	Fine and gross motor subscales	BSID-III at 8 and 24 months	-	No significant main effect in receptive language subscale between waking an nonwaking groups: Estimate (SE) = 0.31 (0.24), t= 1.28, p= 0.202

					waking group (night waking ≥ 3 awakenings) and nonwaking group (≤ 1 awakening)					No significant main effect in expressive language subscale between waking an nonwaking groups: Estimate (SE) = 0.15 (0.25), t= 0.62, p= 0.534
3	Pennestri[15] 2018	Cohort study, Canada	At 6 months : 388, at 12 months : 369	6-12 months	6 or 8 hours of uninterrupted sleep through the night	Self-administered questionnaire asking about the infants' sleep habits at 6 and 12 months	Mental index including cognitive and language development level	BSID-II at 6 and 12 months	Infant's sex, socioeconomic status, breastfeeding status, co-sleeping status, and total sleep duration in a 24-hour period	Association between sleeping through the night or not at 6 and 12 months using the 6-hour criterion and metal development: At 6 months: β = −1.78, p = 0.10 At 12 months: β = −1.19, p = 0.45<br><br>Association between sleeping through the night or not at 6 and 12 months using the 8-hour criterion and mental development: At 6 months: β = −1.53, p = 0.15 at 12 months: β = −0.92, p = 0.50
4	Sun[26] 2018	Cross-sectional study, China	Infant: 590, Toddler : 512	2-30 months	Frequency of night awakenings	BISQ	MDI including cognitive and language development level	BSID-I	Recruited province, child's age and sex, birth weight, maternal	Association between sleep infants' nighttime awakenings and MDI: 1×/night:

									education level, bedroom sharing and being currently breastfed	β±SE: 1.45 ± 2.56, p= 0.57<br>2×/night:<br>β±SE: 6.03 ± 2.43 p= 0.01<br>≥ 3×/night:<br>β±SE: 2.30 ± 2.38, p=0.34<br><br>Association between sleep toddlers' nighttime awakenings and MDI:<br>1×/night<br>β±SE: −0.09 ± 1.79, p= 0.96<br>2×/night<br>β±SE: 0.20 ± 1.99, p= 0.92<br>≥ 3×/night<br>β±SE: −5.15 ± 2.39, p= 0.03
5	Pecora[27] 2022	Cohort study, Italy	156	4-8month	Night awakening number and duration	BISQ	Language Understanding score	MCDI-SF at 8month	Breastfeeding, using a pacifier, siblings, co-sleeping	Correlation between number of night waking at 4month and language -0.18 , p<0.05<br><br>Correlation between duration of night waking at 4month and language:0.02 , p>.05<br><br>Correlation between number of night

										waking at 8month language : -0.18 , p<.05<br><br>Correlation between duration of night waking at 8month and language:-0.02, p>.05
**Motor development**										
1	Mäkelä[23] 2018	Cohort study, Finland	151	8-24 months	Number of night awakenings (between midnight and 6 a.m)<br><br>waking group (night waking ≥ 3 awakenings) and nonwaking group (≤ 1 awakening)	BISQ at 8 months	Fine and gross motor subscales	BSID-III at 8 and 24 months	-	No significant main effect in fine motor subscale between waking an nonwaking groups:<br>Estimate (SE) = 0.22 (0.25), t= 0.88, p= 0.380<br><br>No significant main effect in gross motor subscale between waking an nonwaking groups:<br>Estimate (SE) = 0.22 (0.28), t= 0.79, p= 0.428
2	Pennestri[15] 2018	Cohort study, Canada	At 6 months : 388, at 12 months : 369	6-12 months	6 or 8 hours of uninterrupted sleep through the night	Self-administered questionnaire asking about the infants' sleep habits at 6 and 12 months	Psychomotor index including fine and gross motor development level	BSID-II at 6 and 12 months	Infant's sex, socioeconomic status, breastfeeding status, co-sleeping status, and	Association between sleeping through the night or not at 6 and 12 months using the 6-hour criterion and psychomotor development:

									total sleep duration in a 24-hour period	At 6 months:<br>β = −1.33, p = 0.39<br>At 12 months:<br>β = 1.43, p = 0.49<br><br>Association between sleeping through the night or not at 6 and 12 months using the 8-hour criterion and psychomotor development:<br>At 6 months:<br>β = -2.14, p= 0.15<br>at 12 months:<br>β = −0.29, p = 0.87
3	Sun[26] 2018	Cross-sectional study, China	Infant: 590, Toddler : 512	2-30 months	Frequency of night awakenings	BISQ	PDI including fine and gross motor development	BSID-I	-	No significant differences between four groups (without night awakenings, 1×/night, 2×/night, ≥ 3×/night awakenings) in PDI score
**Growth and anthropometric indices**										
1	Petrov[33] 2021	Cohort study, United states	126	1-36 months	Longest nocturnal sleep bout	BISQ-R at 1 month	RWG, and OW	RWG: >0.67 positive change in weight for age Z-score from birth to 6 months<br><br>OW: BMI percentile ≥85 at 36 months using the Centers for Disease Control and Prevention growth charts	Weight-for-age Z score at birth, months breastfed (measured until 6 month for RWG and 12 month for OW), intervention	Association between longest nocturnal sleep bout and RWG: adjusted OR (95%CI) = 1.19 (0.91-1.55)<br><br>Association between longest nocturnal sleep bout and RWG: adjusted OR (95%CI) = 0.99 (0.74-1.33)

									assignment group, and maternal parity, prenatal body mass index, gestational weight gain, and education attainment	
2	Tikotzky[34] 2010	Cohort study, Israel	96	6 month	Number of night wakings	Actigraphy and BISQ at 6 months	Weight, length, WEFL, and WLR	WEFL: weight above weight expected for length at 6 months, WLR: weight to length ratio at 6 months	-	Pearson correlation between number of night wakings and weight: 0.03, p>0.05<br><br>Pearson correlation between number of night wakings and length: -0.14, p>0.05<br><br>Pearson correlation between number of night wakings and WEFL: 0.12, p>0.05<br><br>Pearson correlation between number of night wakings and WLR: 0.09, p>0.05
3	Wang[16] 2018	Cohort Study, Netherland	2308	6-36 months	Number of the night awakenings	Child's sleep questionnaire at 6,14 and 36 months	BMI z-score	BMI z scores using the World Health Organization Growth Standard at 6, 14 and 36 months	Exact age of the child at sleep measurement, exact age of the child at BMI	Association between night awakenings ≥3 and BMI z scores at 6 months: adjusted β(SE)= 0.075 (0.068), p=0.27

									measurement, maternal age, maternal educational level, maternal pre-pregnancy BMI, and parity, child gender, child ethnic background, child birth weight, gestational age, and duration of breastfeeding, and intervention groups; Model 2 further adjusted for child screen time	Association between night awakenings ≥3 and BMI z scores at 14 months: adjusted β(SE)=-0.048 (0.079), p=0.54<br><br>Association between night awakenings ≥3 and BMI z scores at 36 months: adjusted β(SE)=0.001 (0.134), p=0.99<br>z-score of the children at any ages(6,14,36month)

Table S3- Quality ratings from the NIH's quality assessment tool

Number	Author, year	Quality score	Quality status
1	Hall[28], 2012	64%	Fair
2	Bernier[22], 2010	85%	Good
3	Dione[12], 2011	71%	Fair
4	Hysing[13], 2016	50%	Fair
5	Mäkelä[23], 2018	85%	Good
6	Mäkelä[14], 2020	64%	Fair
7	Mindell[29], 2017	78%	Good
8	Morales-Muñoz[30], 2020	85%	Good
9	Panneastri[15], 2018	78%	Good
10	Petrov[33], 2021	71%	Fair
11	Pisch[24], 2019	85%	Good

12	Plancoulaine[25], 2017	85%	Good
13	Sun[26], 2018	575	Fair
14	Tikotzky[34], 2010	57%	Fair
15	Wang[16], 2019	78%	Good
16	Zaidman-Zait[31], 2015	85%	Good
17	Makela[32], 2021	85%	Good
18	Pecora[27], 2022	85%	Good
